# Detection of positively remodeled coronary artery lesions by multislice CT and its impact on cardiovascular future events

**DOI:** 10.1186/s43044-019-0029-8

**Published:** 2019-11-21

**Authors:** Haitham Galal, Tarek Rashid, Wesam Alghonaimy, Diaa Kamal

**Affiliations:** Department of Cardiology, Ain Shams University, Cairo, Egypt

**Keywords:** MSCT CA, CA remodeling

## Abstract

**Background:**

Positive arterial remodeling may be a characteristic of early proliferative lesions. The study was done to identify the different morphological characteristics of the positively remodeled coronary lesions, and causing non-significant arterial stenosis, as detected by multislice computed tomography coronary angiography (MSCT CA) and its predictors of cardiovascular clinical events at 90-day follow-up. The study included 55 patients who were candidate for MSCT CA and found to have a single-vessel disease with less than 70% stenosis positively remodeled lesions. The most expansive or solitary lesion was selected for each patient. Positive remodeling defined as remodeling index (RI) > 1.05. We followed the patients clinically for 90 days.

**Results:**

Twenty-four patients had a history of acute coronary syndrome at initial presentation with normal LV systolic function for all studied patients. Dyslipidemia was found in 37 patients (67.3%) while diabetes was found in 29 patients (52.7%). The majority of the lesions were found in the proximal LAD (43.6%). The mean calculated remodeling index was 1.41 ± 0.25. At the end of 90 days, 25 patients had clinical events in the form of unstable coronary syndromes, coronary interventions, or coronary angiography related to the index lesion. The predictors of clinical events were duration of DM, higher degree of luminal narrowing, calculated wall/lumen area percentage, plaque burden, plaque-specific calcification, and total calcium score at remodeling site as well as a lower percentage of low-attenuation plaque area. The mean calculated wall/lumen area percentage was 263.72 ± 122.71%. A cut-off value of > 226% was found a predictor for clinical events. The mean plaque burden percentage was 69.72 ± 9.71%, a value of > 69% was found a predictor for clinical events. Both values had a sensitivity of 68% and specificity of 86.6% and PPV of 81%. Positively remodeled lesions with a high RI > 1.4 were correlated with patients who had acute coronary syndrome on their initial presentation.

**Conclusion:**

Different morphological characteristics of positively remodeled non-occlusive atherosclerotic plaques as detected by multislice CT coronary angiography may be good potential predictors of future cardiovascular events.

## Background

Clinical researches showed that some of the coronary plaques are suddenly activated and rupture, causing acute events, whereas others remain asymptomatic [1].

About two thirds of acute coronary events are associated with atheromatous plaque disruption. Unstable plaques are characterized by large volumes and large necrotic cores and covered by thin fibrous cap. Low-density and non-calcified plaques were found to be the strongest predictor of cardiac events, regardless of lesion severity, and are potential markers for plaque vulnerability. Multislice coronary angiography (MSCA) may be an effective diagnostic tool for patients with chest pain who have a normal ECG and cardiac enzymes in the emergency department. MSCA could analyze coronary plaques both quantitatively and qualitatively [2].

The vessels at the site of plaque disruption show positive remodeling. In addition, small calcific concretions in fibrous caps may contribute to plaque instability. Vulnerable plaques are termed thin cap fibroatheroma (TCFA) [3].

The coronary arterial remodeling was defined as a change in the vessel diameter at the plaque site in comparison with the reference segment set proximal to the lesion in a normal appearing vessel segment (reference segment). When the diameter at the plaque site exceeds by at least 10% the reference segment, this is called the remodeling index calculated by MSCT*.* The most important determinants of the plaque vulnerability are plaque size and necrotic core size. In particular, lesions with hemorrhage, large necrotic cores, lipid core, macrophage inflammation, and calcification are more likely to show a positive vascular remodeling. Stable plaques show no positive remodeling [3]. Plaque progression and regression are associated with arterial expansion (positive remodeling) and shrinkage (negative remodeling), respectively. Sudden changes of mildly stenotic lesions with regions of positive arterial remodeling are the cause of acute coronary syndrome in many studies [1]. In conclusion, positively remodeled lesions on MSCT are associated with plaque vulnerability on virtual histology intravascular ultrasound (VH IVUS) images with a higher necrotic core and a higher prevalence of TCFA [4]. This study was done to identify the different morphological characteristics of the positively remodeled coronary lesions, and causing non-significant arterial stenosis, as detected by multislice computed tomography coronary angiography (MSCT CA) and its predictors of cardiovascular clinical events at 90-day follow-up.

## Methods

Our Study was conducted on 55 patients who were presented to the International Cardio Scan Center to undergo multislice CT CA according to the SCCT guidelines’ appropriateness criteria. Those patients who were found to have a single-vessel disease with lesions showing positive arterial remodeling causing less than 70% stenosis were selected. A single lesion for each patient was chosen which was either the solitary lesion or the most expansive one.

### Exclusion criteria

Exclusion criteria are pregnant females, patients with renal impairment, patients who are hemodynamic unstable, patients sensitive to contrast media, patients with marked irregular rhythm, patient who had previous PCI or CABG, lesions causing more than 70% stenosis, and high total and lesion-specific calcium score hindering proper plaque assessment.

### History

Thorough history taking was done to evaluate initial presentation (stable angina, unstable angina, atypical chest pain, dyspnea), cardiovascular risk factors as smoking, DM, hypertension, and dyslipidemia, and demographic data including age, sex, and BMI.

### Echocardiography

This was done for all patients to assess mainly the LV systolic function by using the Simpson^’^s method.

### MSCT

MSCT was performed using a 64 slice CT machine (Aquillion 64, Toshiba Medical, Tokyo, Japan). Fifty milliliters of contrast agent (Ultravist) was injected intravenously as a bolus with a rate of 4.0 mL/s. Acquisition of the computed tomography (CT) data and the electrocardiogram (ECG) trace were started as the signal density level in the ascending aorta reached a predefined threshold of 100 Hounsfield units (HU). The volume data set for coronary artery imaging was acquired in spiral mode, with a collimation of 64 × 0.5 mm, a gantry rotation of 400 ms, helical pitch of 3.2, tube energy of 120 kV, and an effective tube current of 400 mA. ECG was monitored continuously during image acquisition. The scan data were reconstructed using a segment algorithm and transferred to a computer workstation (Vitrea, Tokyo, Japan) for post processing.

### Coronary artery calcification (CAC)

Calcification was quantified on a workstation (Vitrea, Tokyo, JAPAN) with scoring software. CAC was defined on CT images as the presence of more than two contiguous pixels with greater than 130 HU. The CAC score in each lesion was computed by the Agatston method.

### Assessment of plaques

After visual assessment [5] of the volume-rendered images, the coronary artery plaques were examined on both the axial and curved multiplanar reconstruction images. For every patient, we selected the most expansive plaque causing less than 70% stenosis in a vessel with reference diameter > 2 mm.

#### Plaque evaluation and coronary remodeling by MSCT

The remodeling index (RI) was defined as the ratio between the vessel area at the lesion segment/vessel area at the reference segment with positive remodeling showing RI > 1.05. The acquired images were transferred to offline workstation (Toshiba Vitrea Medical Systems).

#### The results of the CT will be assessed regarding

##### Post processing

Serial multiplanar reconstructions (slice thickness 1 mm) were rendered in an orientation perpendicular to the longitudinal axis of the respective coronary artery segment. The cross-sectional vessel area was calculated in a reference segment with no detectable plaques proximal to it and close to the respective coronary lesion. Several measurements were done at the site of maximum arterial remodeling and compared with the reference segment using the automated software for vessel analysis. (Vessel Analysis, Vitrea, Toshiba Medical) including lumen area, vessel surface area, wall area, wall/lumen area percentage, total plaque area, and plaque burden percentage.

Each plaque was analyzed separately and the relative component (low-attenuation, medium attenuation, and calcific components) were measured by both surface area and area percentage together with mean density of the plaque. The software differentiated each plaque component into three categories according to the following color-coded spectrum and HU attenuation.

- *Plaque 1:* Low-attenuation plaque exhibiting attenuation range from (− 100:49 HU coded with red color)
- *Plaque 2:* Medium attenuation plaque exhibiting attenuation range from (50:149 HU) coded with blue color.
- *Plaque 3:* High-attenuation plaque with attenuation range from (150:1300 HU) coded with yellow color corresponding to calcium.

The cross-sectional vessel areas were the tool to measure the remodeling index (RI = affected segment surface area/reference segment surface area) and the remodeling index was calculated. The severity of all lesions included was causing less than 70% stenosis exhibiting a range of minimal, mild, moderate stenosis according to the SCCT guidelines and CAD RADS Reporting System (10.1016/j.jcmg.2016.05.005).

A 3-month follow-up was done for possible cardiovascular events related to the index lesion including hospitalization with ACS, heart failure, myocardial infarction, coronary angiography, and PCI.

### Statistical analysis

The Statistical Package for Social Science (IBM SPSS) version 20 was used for statistical analysis. Quantitative data were presented as mean, standard deviations, and ranges while qualitative data were presented as number and percentages. Chi-square test and/or Fisher exact test was used for comparison between two groups with qualitative data. Fisher exact test was used instead of chi-square test when the expected count in any cell was found less than 5. Independent *t* test was used for comparison between two independent groups regarding their quantitative data with parametric distribution.

## Results

It was a prospective sinfle-center study that included 55 patients who were presented to the International Cardio Scan Center for CCTA (coronary computed tomography angiography). The study included 39 male patients (70.9%). The mean age was 55.62 ± 7.78 years.

Most of the patients, 24 had history of CCU admission as a case of ACS on their initial presentation, 8 patients had stable coronary artery disease while 14 patients were classified to have atypical chest pain, and 9 patients with dyspnea. The mean ejection fraction among them was 59.33% ± 5.62%, indicating a normal LV systolic function for all patients. Among different coronary artery disease risk factors, dyslipidemia was found in the majority 37 patient (67.3%). Diabetes was found in 29 patients (52.7%), hypertension in 31 patients (56.4%) as shown in Table 1.

**Table 1:** Patient demographic data, risk factors, and clinical presentation

		No. = 55
Age (years)		55.62 ± 7.78
Males		39 (70.9%)
BMI		28.25 ± 2.94
Smokers		29 (52.7%)
Hypertensive		31 (56.4%)
Diabetics		29 (52.7%)
DM type	DM type I	4 (14.3%
	DM type II	24 (85.7%)
Duration of DM (years)		14.39 ± 5.20
Dyslipidemic		37 (67.3%)
Family history		10 (18.2%)
Initial presentation	ACS	24 (43.6%)
	Atypical chest pain	14 (25.5%)
	Dyspnea	9 (16.4%)
	Stable angina	8 (14.5%)
EF (%)	59.33 ± 5.62	

*BMI* body mass index, *DM* diabetes mellitus, *EF* ejection fraction, *ACS* acute coronary syndrome

### CT vessel analysis and plaque characterization (Table 2)

A total of 192 lesions were examined in proximal and mid segments of the coronary arteries. Either solitary or the most expansive lesion for each patient was chosen. Final 55 lesions with positive remodeling causing less than 70% stenosis by visual assessment [5] were included. The non-obstructive positively remodeled lesions by CT were classified by visual assessment, stenosis grading according to SCCT guidelines 2014, into minimal (< 24% stenosis), mild (25–49%), and moderate (50–69% stenosis).

**Table 2:** CT measurements at both reference and remodeling segments

	Variable	Reference segment	Remodeling segment
Area (mm^2^)	Vessel	18.93 ± 6.98	26.49 ± 10.63
	Wall	8.40 ± 3.37	17.49 ± 6.92
	Total plaque	8.44 ± 3.28	17.22 ± 6.64
	Low-attenuation plaque	*Median* 2 (2–3) *1–28*	5.00 (4–6) *2–10*
	Medium attenuation plaque	*Median* 5 (4–6) 2–71	8.00 (6–10) 3–21
	High-attenuation plaque	*Median* 0 (0–1) 0–6	2 (1–5) 0–13
Percentage (%)	Wall/lumen area	82.85 ± 19.00	222.82 ± 98.19
	Plaque burden	44.67 ± 5.84	66.80 ± 8.29
	Low-attenuation plaque area	29.76 ± 9.59	32.73 ± 11.04
	Medium attenuation plaque area	62.76 ± 9.24	50.04 ± 14.53
	High-attenuation plaque area	*Median* 3 (0–10) 0–34	9 (5–24) 0–62
Mean density (HU)	Low-attenuation plaque	*Median* 18 (13–26) 0–30	6 (0–16) − 21–21
	Medium attenuation plaque	92.04 ± 9.79	94.18 ± 7.05
	High-attenuation plaque	*Median* 158 (122–170) 0–230	185 (162–230) 0–630
Calcium score (Agatston method)	Plaque-specific	*Median* 0 (0–0) 0–58	0 (0–0) 0–91
	Total CA score	*Median* 0 (0–0) 0–850	0 (0–0) 0–850

*HU* Hounsfield unit

The majority of the positively remodeled lesions in this study were found to be exhibiting moderate degree of stenosis in 24 lesions (43.6%) out of total 55 lesions. Most of the chosen lesions were found in the proximal LAD presenting 52.7% and mid LAD (30.9%). The RCA remodeled lesions were found more in the mid segment (7.3%) compared to the proximal RCA (1.8%). There was no difference between proximal and mid LCX lesions prevalence.

Semi-automated calculations were done for each plaque to identify the 3 different types of plaque components (low, medium, and high-attenuation plaques). The calculated remodeling index mean was 1.41 ± 0.25. CT measurements were done at both the reference segments and remodeling segments respectively (Table 2).

### Follow-up

**Fig. 1:**
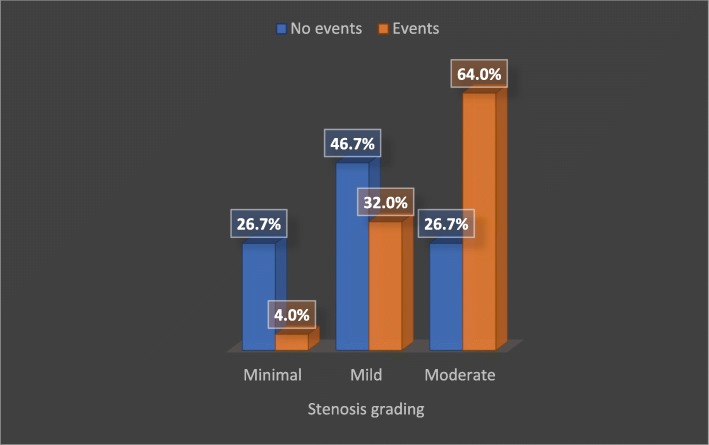
Stenosis grading in relation to clinical events

A 90-day follow-up was conducted to assess adverse cardiovascular events. Total 25 patients were found to have events: 3 patients were hospitalized as ACS and treated medically, 6 patients were hospitalized as ACS and had CA, 10 patients had CA and were set for medical treatment, and 6 patients had PCI related to the index lesion. There was no myocardial infarction nor death during the follow-up period. Duration of DM and degree of luminal narrowing was found to be significant predictors of future clinical events as shown in Table 3 and Fig. 1.

**Table 3:** Relation between different cardiovascular risk factors and cardiovascular events

		No events	events	*P* value
		No. = 30	No. = 25	
Age (years)		55.17 ± 7.91	56.16 ± 7.74	0.642
Males		20 (66.7%)	19 (76.0%)	0.448
BMI		27.77 ± 2.93	28.84 ± 2.90	0.180
Smoking	Nonsmoker			0.657
	Smoker	15 (50.0%)	14 (56.0%)	
Hypertensive		16 (53.3%)	15 (60.0%)	0.620
Diabetics		15 (50.0%)	14 (56.0%)	0.657
DM type	DM type I	2 (13.3%)	2 (15.4%)	0.877
	DM type II	13 (86.7%)	11 (84.6%)	
Duration of DM		*12.60 ± 3.09*	*16.46 ± 6.41*	0.048*
Dyslipidemia		19 (63.3%)	18 (72.0%)	0.495
EF (%)		60.50 ± 5.73	57.92 ± 5.25	0.090

*BMI* body mass index, *DM* diabetes mellitus, *EF* ejection fraction

*Significant *P* value

Regarding correlation of future cardiovascular clinical events and CT vessel measurements and plaque characterization at the remodeling site, larger area of high plaque attenuation, higher lumen/wall area percentage, higher plaque burden, higher plaque-specific calcification, and higher total calcium score together with a lower percentage of low-attenuation plaque area were significantly correlated with the presence of future clinical events as shown in Table 4.

**Table 4:** Relation between different CT vessel measurement and plaque characterization with future cardiovascular clinical events

Variable at remodeling site		No clinical events No. = 30	Clinical events No. 25	*P* value
Area (mm^2^)	Vessel	25.30 ± 10.68	27.92 ± 10.60	0.368
	Wall	16.27 ± 7.11	18.96 ± 6.51	0.152
	Total plaque	15.93 ± 6.65	18.76 ± 6.42	0.117
	Low-attenuation plaque	Median 5 (4–6) 2–10	5 (4–6) 2–9	0.421
	Medium attenuation plaque	Median 7 (6–10) 3–15	8 (7–10) 4–21	0.288
	High-attenuation plaque	Median 1 (0–2) 0–13	2 (1–6) 0–12	0.025*
Percentage (%)	Wall/lumen area	188.73 ± 53.34	263.72 ± 122.71	0.004**
	Plaque burden	64.37 ± 6.03	69.72 ± 9.71	0.016*
	Low-attenuation plaque area	36.60 ± 10.64	28.08 ± 9.81	0.003**
	Medium attenuation plaque area	50.87 ± 13.02	49.04 ± 16.38	0.647
	High-attenuation plaque area	Median 9 (1–11 0–37	9 (6–41) 0–62	0.117
Mean density (HU)	Low attenuation	Median 6 (1–14) − 21.00–20	6 (0–16) − 19.00–21	0.806
	Medium attenuation	93.23 ± 7.32	95.32 ± 6.67	0.278
	High attenuation	Median 170 (162–208) 0–428	196 (166–381) 0–630	0.106
Calcium score (Agatston method)	Plaque-specific	Median 0 (0–0) 0–18	0 (0–62) 0–91	0.010*
	Total calcium score	Median 0 (0–0) 0–48	0 (0–260) 0–850	0.009**
Remodeling index		1.39 ± 0.21	1.44 ± 0.30	0.479

*HU* Hounsfield unit

*Significant *P* value, **Highly significant *P* value

**Fig. 2:**
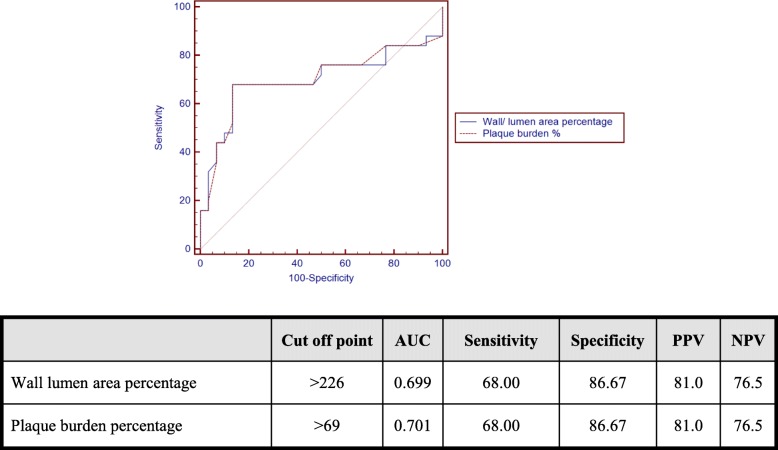
ROC curve showing cut-off value for the wall lumen area percentage and plaque burden percentage

The calculated wall/lumen area percentage at remodeling site mean was 263.72 ± 122.71%. A ratio cut of value of > 226% was found a predictor for clinical events. The plaque burden percentage mean was (69.72 ± 9.71%), and a value of > 69% was found a predictor for clinical events. Both values had a sensitivity of 68, specificity of 86.6, and PPV of 81 as shown in the ROC curve (Fig. 2).

### Remodeling index

There was no significant correlation between calculated remodeling index and different coronary artery disease risk factors nor the site of the remodeled lesions. Both lumen area and vessel area at remodeling site were significantly higher among those with higher calculated RI. Patients with high RI > 1.4 had significantly a higher area of lower attenuation plaque component (mm^2^). However, the area of medium attenuation plaque component (mm^2^) was not associated with high RI. Patients who had acute coronary syndrome on their initial presentation were found to have a correlation with positive remodeled lesions with high RI > 1.4.

## Discussion

Despite plaque accumulation, expansion (positive remodeling) of early lesions maintains the lumen size. In contrast, shrinkage (negative remodeling) contributes to luminal stenosis independent of plaque accumulation [1].

In the present study, the most expansive non-obstructive atherosclerotic lesion was identified. The reference segment was identified as the most proximal segment without detectable plaque and as close as possible to the lesion. We found that positive arterial remodeling was present in both genders, and there was a change in the vessel size and substantial increase in the outer contour of the vessel wall. Patients who were presented with unstable angina had a significant association with lesions with higher remodeling index. The mean remodeling index was 1.4, and low-attenuation plaque area was found larger at the site of remodeling with a mean of 32.73 ± 11.04% of the whole plaque area compared to the reference segment.

Medium attenuation plaque component area was found larger at the reference segment compared to the remodeling site with a mean of (62.76 ± 9.24% vs 50.04 ± 14.53%).

All patients had normal LV systolic function. Among different cardiovascular risk factors, dyslipidemia was the most common (67.3%), diabetes was present in 52.7% patients mainly type 2 DM. Most of the lesions included in the study were exhibiting 40–50% luminal stenosis by visual assessment. A significant association was present between low-attenuation plaque in lesions with high remodeling index. Remodeling was found mainly in the LAD 83.6% affecting more the proximal segments. At remodeling site, the mean vessel area was 26.49 ± 10.63 mm^2^, mean wall area 17.49 ± 6.92 mm^2^, mean plaque burden 66.80 ± 8.29%, and mean area of the plaque 17.22 ± 6.64. At the reference site, the mean vessel area was 18.93 ± 6.98 mm^2^, mean wall area 8.40 ± 3.37 mm^2^, mean plaque burden 44.67 ± 5.84%, and total area of the plaque 8.44 ± 3.28 mm^2^.

These findings were similar to the results conducted by Schmid et al. [6]. In their study, they found that lipid rich plaques with low attenuation on CT were correlated with positive remodeling and subsequently increased risk for plaque rupture and clinical events. In contrast to our mean remodeling index of 1.4, their mean remodeling index was found 1.17 ± 0.30 and this could be attributed to the majority of their studied lesions which were located in the mid segments of the vessels. Forty-seven lesions (42%) were located in proximal segments and 65 (58%) in mid coronary segments. In both studies, the remodeling index was not significantly associated with any of the coronary risk factors, lesion location, or statin use. The mean cross-sectional vessel area in the lesion was 0.25 ± 0.08 cm^2^, and the mean reference vessel area was 0.22 ± 0.09 cm^2^. The mean CT attenuation of atherosclerotic plaque in the lesions was 71 ± 26 HU.

The mean attenuation measured in positively remodeled lesions was significantly lower than in lesions with no or negative remodeling.

The same results were found by Pflederer and his colleagues [7] that in patients with acute coronary syndrome, the incidence of positively remodeled culprit lesions was more than those with stable coronary artery disease. The remodeling index and the plaque and media complex area in the acute coronary syndrome group were also larger than those in the stable angina group.

Similarly, a study was done by Motoyama et al. [3], who studied the CT characteristics of coronary lesions in ACS. He compared between 38 patients with ACS and 33 patients with stable angina, and the coronary plaques were evaluated in both arms using CT plaque characteristics, including vessel remodeling and consistency of non-calcified plaque. They found that positive remodeling (87% vs. 12%), non-calcific plaques (NCP) less than 30 HU (79% vs. 9%), and spotty calcification (63% vs. 21%) were significantly more frequent in the ACS patients.

In our study, after 90-day follow-up, we found that majority of patients were just allocated to medical treatment with no adverse future cardiovascular events in 51.8% and those who developed clinical events were 48.2%. Duration of diabetes and higher degree of luminal stenosis by visual assessment > 60% in the studied lesions were more associated with future cardiovascular events. We identified each plaque similar to IVUS into 3 different color codes using automated software. We were able to identify plaque area instead of plaque volume. However, the duration of follow-up was short, but we ended with the same conclusion to Nadjiri et al. and Tesche et al. [8, 9] that high-risk plaque characteristics including positive remodeling and low-attenuation plaque area were associated with future cardiovascular clinical events.

Moreover, we found that the higher plaque burden and higher wall/lumen area percentage at site of positive remodeling were significantly associated with future adverse clinical events.

Nadjiri et al. [8] studied the prognostic value of quantitative plaque assessment in coronary CT angiography during 5 years follow-up of 1168 patients. MACE was present in 46 patients (3.9%). MACE was associated with all plaque characteristics, and the strongest association was observed for low-attenuation plaque volume (LAPV).

Tesche et al. [9] studied the prognostic implications of coronary CT angiography-derived quantitative markers for the prediction of major adverse cardiac event. He found that patients with MACE had significantly more obstructive coronary lesions with higher non-calcific plaque volume (NCPV) (67.3 mm^3^ vs. 56.1 mm^3^), plaque burden (66.3% vs. 44.9%), and greater lesion length.

Yamamoto et al. [10] concluded that identification of non-calcified atherosclerotic lesions (NCALs) with low-attenuation plaques (LAP) and positive remodeling (PR) characteristics by CT coronary angiography indicate additional prognostic information to coronary stenosis for the prediction of future coronary events.

The long duration of follow-up in the previous studies influenced the kind of clinical events occurred. Hard events like death and myocardial infarction occurred in their MACE groups were not found among our patients who were followed up for shorter duration.

Previous studies showed that positively remodeled lesions detected by CT coronary angiography were associated with increased levels of plaque vulnerability on VH IVUS images. Thus, evaluation of remodeling on CT coronary angiography may provide a valuable marker for plaque vulnerability. A head to head comparison of CT coronary angiography with IVUS confirms the diagnostic accuracy of CCTA in the quantitative assessment of coronary plaques as shown by Nakazato et al. [11] who observed a high correlation between total plaque volumes as quantified by CCTA in comparison to IVUS with no significant differences between the two methods. Furthermore, CCTA had high diagnostic accuracy for identification of adverse plaque characteristics (low-attenuation plaque, positive remodeling, and spotty calcification) with no statistical differences with IVUS. In summary, CCTA allows quantitative analysis of plaque vessel area, lumen area, and plaque burden in addition to accurate detection of coronary atherosclerotic plaques.

## Conclusions

Different morphological characteristics of positively remodeled non-occlusive atherosclerotic plaques as detected by multislice CT coronary angiography may be good potential predictors of future cardiovascular events.

### Limitations

1. It was a single center study.
2. Small sample size.
3. Short-term follow-up.
4. No hard events were detected during follow-up (infarction or death).
5. Although we did all our effort meticulously to assure that patients who underwent coronary intervention during the follow-up period were done due to new coronary events and not due to MSCT results, still the results were driven in the first 3 months of follow-up, the period which is usually excluded from most of other studies to avoid this confusion.

## Data Availability

The datasets used and analyzed during the current study are available from the corresponding author on reasonable request.

## Abbreviations

CAD: Coronary artery disease
CCTA: Coronary computed tomography angiography
LAP(V): Low-attenuation plaques (volume)
MDCT: Multidetector computed tomography
MSCT CA: Multislice computed tomography coronary angiography
NCALs: Non-calcified atherosclerotic lesions
NCP: Non-calcific plaques
NCPV: Non-calcific plaque volume
PR: Positive remodeling
SCCT: Society of cardiovascular computed tomography
TCFA: Thin cap fibroatheroma
VH IVUS: Virtual histology intravascular ultrasound
